# Immunosuppressive Roles of Galectin-1 in the Tumor Microenvironment

**DOI:** 10.3390/biom11101398

**Published:** 2021-09-23

**Authors:** Yanyu Huang, Hsiao-Chi Wang, Junwei Zhao, Ming-Heng Wu, Tsung-Chieh Shih

**Affiliations:** 1 Department of Biochemistry and Molecular Medicine, University of California Davis, Sacramento, CA 95817, USA; yyahuang@ucdavis.edu (Y.H.); juwzhao@ucdavis.edu (J.Z.); 2 Department of Internal Medicine, University of California Davis, Sacramento, CA 95817, USA; 69kikiwang@gmail.com; 3 Graduate Institute of Translational Medicine and International Ph.D. Program for Translational Medicine, College of Medical Sciences and Technology, Taipei Medical University, Taipei 11031, Taiwan; mhwu1015@tmu.edu.tw

**Keywords:** Galectin-1, immunotherapy, microenvironment, LLS30

## Abstract

Evasion of immune surveillance is an accepted hallmark of tumor progression. The production of immune suppressive mediators by tumor cells is one of the major mechanisms of tumor immune escape. Galectin-1 (Gal-1), a pivotal immunosuppressive molecule, is expressed by many types of cancer. Tumor-secreted Gal-1 can bind to glycosylated receptors on immune cells and trigger the suppression of immune cell function in the tumor microenvironment, contributing to the immune evasion of tumors. The aim of this review is to summarize the current literature on the expression and function of Gal-1 in the human tumor microenvironment, as well as therapeutics targeting Gal-1.

## 1. Introduction

In 2020, the range of newly diagnosed cancer incidences was about 19.3 million globally, resulting in about 10 million fatalities. Owing to the world’s increasing population, if the number of incidences continues at this rate, the number of cancer cases worldwide will increase to 28.4 million by the year 2040 [1]. All cancers share the same characteristics in that they are genetic disorders caused by DNA mutations, with most pathogenic mutations either being induced by exposure to mutagens or occurring spontaneously as part of aging. Genetic alterations are heritable, and as a result the cells harboring these aberrations are subject to Darwinian selection. Immunologically, tumor cells can be regarded as modified self-cells that have eluded typical growth-regulating machinery [2]. The evasion of immune surveillance is an accepted hallmark of tumor progression [3], and treatment strategies for targeting immune-suppressive pathways have led to enhanced patient survival in multiple cancers [4]. 

Cell surface carbohydrates carry out a vast array of roles and are imperative to normal cellular physiology. In addition to serving as ligands of glycan-binding proteins (GBPs), they directly impact glycoprotein function by enabling glycan-dependent signaling on the cell surface. Carbohydrate interactions on the GBP cell surface play key roles in immune responses and in the tumor microenvironment [5]. GBPs were first discovered by Ashwell and Morrell in the 1960s and were designated as vertebrates (the asialoglycoprotein receptor) [6]. In 1975, Teichberg et al. discovered electrolectin from the electric eel (*Electrophorus electricus)* [7]. It was the first vertebrate galectin, Galectin-1 (Gal-1). Shortly after that, Gal-1 was isolated in 1976 by Kornfeld et al. from extracts of calf heart and lung [8]. In the same year, Barondes et al. isolated a similar galectin from chick muscle extracts [9]. To date, 15 different galectins have been discovered in mammals, with 11 found in humans, all in subunit size (14–39 kDa) [10].

Galectin expression varies from cell to cell, as it relies on the state of activation of a specific cell. All cellular types express at least one galectin, with different galectins being expressed in high concentrations in different cell types. After synthesis in the cytosolic ribosomes, they are translocated to the nucleus or additional subcellular locations. Galectins are lacking in secretion signal peptides, archetypal transmembrane segments, and N-termini with acetyl groups with features similar to those of other cytosolic proteins [11]. The anomalous expression of galectins is linked to the incidence, advancement, and metastasis of cancers. Galectins also have a broad spectrum of effects on diverse immune cells, promoting inflammation or inhibiting immune responses mediated by T-cells and dependent on the receptors in specific target cells [11]. Gal-1 has been the most studied galectin since it first displayed hemagglutinating activity in 1975. In recent years, Gal-1 has been known to promote cancer cell growth; in addition, tumor-secreted Gal-1 is involved in immune escape by tumors, indicating that Gal-1 is a critical molecular target in cancer and could be a potentially therapeutic target for cancer treatment.

## 2. Gal-1 Molecular Structures and Biological Functions in Human Cancers

Gal-1 is a 14-kDa lectin encoded by the gene LGALS1 at 22q13.1 [12]. Gal-1 is a galectin family member with an affinity for β-galactosides [13]. Gal-1 folding involves a β-sandwich consisting of two antiparallel β-sheets of five (F1–F5) and six (S1–S6a/b) strands [14]. The N and C termini of each monomer are positioned at the dimer interface, and the glycan-binding sites are located at opposite ends of the dimer. Human Gal-1 exists as a dimer, which is maintained by hydrophobic interactions through the hydrophobic core [14]. This solid hydrophobic core is formed by hydrophobic side chains of Leu4, Ala6, Ile128, Val131, and Phe133 from both subunits [15]. The backbones of residues of Val5, Ser7, Val131, Lys129, and Phe133 from both subunits establish a well-defined hydrogen bond network [15]. The presence of six cysteine residues in the Gal-1 sequence makes it sensitive to oxidation, which limits its physiological activity [16].

In addition to β-galactoside binding activity, Gal-1 participates in protein–protein interaction to regulate a wide range of signaling pathways, particularly the oncogenic pathway. Paz et al. showed that intracellular Gal-1 stabilizes activated H-Ras (G12V) at the plasma membrane, which is essential for inducing the Ras oncogenic signaling pathway [17]. Patterson et al. found that Gal-1 interacts with Gemin4 and is co-immunoprecipitated with the nuclear SMN complexes for the splicing of pre-mRNA [18,19]. Ose et al. found that galectin-1 interacts with Protocadherin-24 and is retained at the plasma membrane. This results in the suppression of the β-catenin signaling by the localization of β-catenin at the plasma membrane [20]. A high expression of Gal-1 has been found in many human cancers [21,22,23,24,25], and Gal-1 mechanisms in cancer progression have been provided in recent reviews [10,26,27].

## 3. Gal-1 in T-Cell Immunodeficiency Diseases 

**Figure 1:**
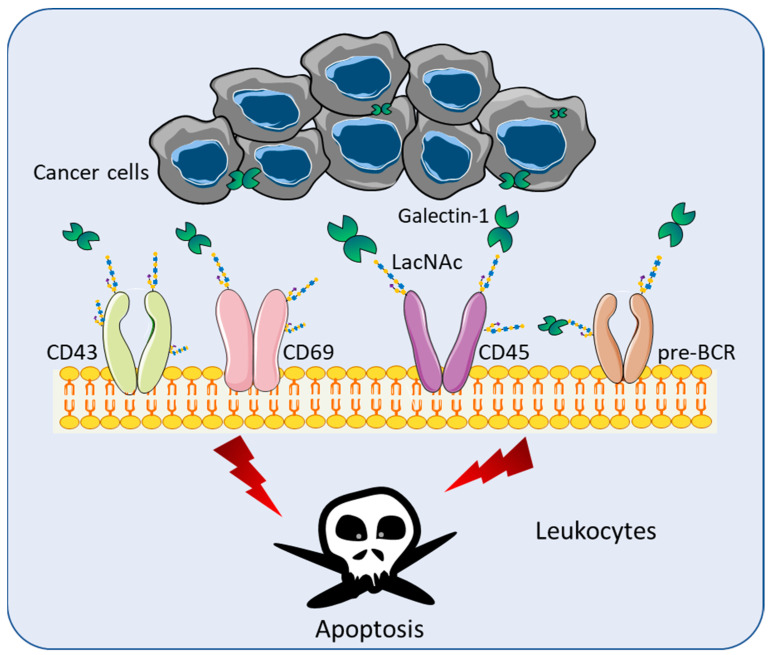
Immunosuppressive functions of Gal-1 in the tumor microenvironment. Gal-1 recognizes terminal galactose residues β-1,4-linked to LacNAc, which is present in different cell receptors including CD43, CD69, CD45, and pre-BCR. Through the binding of LacNAc, Gal-1 can stimulate the apoptosis of effector leukocytes.

Although Gal-1 lacks a secretion signal peptide or archetypal transmembrane segments, Gal-1 is secreted and found in the extracellular space [26]. Gal-1 recognizes terminal galactose residues β-1,4-linked to N-Acetyllactosamine (LacNAc), which are present in the branch of *O-* or *N*-linked glycans on an extensive array of cell receptors including pre-BCR, CD43, CD45, CD69, and vascular endothelial growth factors (VEGF) [27] (Figure 1). Through the binding of LacNAc, Gal-1 can stimulate the apoptosis of effector leukocytes [28] (Figure 1). Various studies have demonstrated that Gal-1 mediates T cell apoptosis through multiple mechanisms, including the loss of mitochondrial membrane potential [29], the activation of the Lck/ZAP-70 signaling pathway [30], the release of cytochrome c [31], the activation of the c-Jun/AP-1 pathway, and the downregulation of Bcl-2 protein expression [32]. The binding of Gal-1 is halted by the modification of LacNAc by the α2,6 sialyltransferase 1 (ST6GAL1), which adds α2,6-linked sialic acid to the terminal galactose of N-linked glycans [27].

Toscano et al. showed that T helper type 2 (Th2) cells were protected from Gal-1 induced cell death through the differential sialylation of cell surface glycoproteins [33]. Consistent with these findings, the treatment of mice with recombinant Gal-1 (rGal-1) has been reported to block the development of T helper type 1 (Th1) cell-mediated diseases [34,35,36]. In broad terms, Th1 cells promote a cellular immune response and Th2 cells produce a humoral immune response [37]. Rabinovich et al. showed that an injection of syngeneic DBA/1 fibroblasts engineered to secrete Gal-1 was able to abrogate clinical and histopathological manifestations of arthritis, and this effect was reproduced by the daily administration of rGal-1 [34]. The cytokine profiles of draining lymph node cells in mice sera showed the inhibition of the proinflammatory response and skewed towards Th2 immunity [34]. Santucci et al. showed that rGal-1 exerts therapeutic activity in Th1-mediated experimental colonic inflammation by eliminating the uncontrolled Th1 response to the hapten [35]. In experimental autoimmune uveitis (EAU), Toscano et al. showed that rGal-1 treatment was sufficient to suppress clinical ocular pathology, inhibit leukocyte infiltration, and counteract pathogenic Th1 cells [36]. The administration of rGal-1 modulates the Th1/Th2 balance toward nonpathogenic Th2 and T-regulatory cytokine profiles [36]. These studies evidenced that rGal-1 suppressed Th1-dependent responses and increased T cell susceptibility to activation-induced cell death.

## 4. Role of Galectins in Cancer Immune Surveillance

In the tumor microenvironment, Gal-1 plays a major role in tumor immune evasion. The mechanisms of Gal-1-mediated tumor immune escape are discussed in the following sections.

### 4.1. Lymphoma

There are two main types of lymphoma: classical Hodgkin lymphoma (cHL) and non-Hodgkin lymphoma (NHL). cHL contains a particular type of cell known as a Reed–Sternberg (RS) cell, which is an abnormal B lymphocyte [38]. NHL cases do not contain Reed–Sternberg cells, and arise from a defect in B cells that express membrane-bound CD20 [39]. In cHL research, Juszczynski et al. found that cHL RS cells overexpressed Gal-1 through an AP1-dependent enhancer [40]. In co-cultures of activated T cells and cHL RS cells, the inhibition of RS Gal-1 via siRNA increased T cell viability and restored the Th1/Th2 balance. In addition, the Gal-1 treatment of activated T cells fostered the secretion of Th2 cytokines and the expansion of CD4^+^CD25^high^FOXP3^+^ T regulatory (Treg) cells [40]. Based on these findings, Rodig et al. tested whether the coordinate expression of activated AP1 pathway components and Gal-1 served as a diagnostic signature of cHL [41]. The immunohistochemical results showed that Gal-1 was selectively expressed by malignant RS cells in 92% (66 of 72 cases) of primary cHLs and that Gal-1 expression was concordant with the activated AP1 component, c-Jun. In contrast, diffuse large B-cell lymphoma, primary mediastinal large B-cell lymphoma, and nodular lymphocyte-predominant Hodgkin lymphoma (another Hodgkin-related entity) do not express Gal-1 [41]. 

In NHL research, Lykken et al. showed that Human NHLs expressed elevated Gal-1 compared with nonmalignant lymphocytes and that Gal-1 expression by lymphoma cells abrogated CD20 immunotherapy in mice. Mechanistically, both exogenous rGal-1 and lymphoma-derived Gal-1 impaired mAb-dependent lymphoma phagocytosis by macrophages in vitro, demonstrating that extracellular Gal-1 can impede macrophage activation and function [42].

### 4.2. Head and Neck Cancer (HNC)

HNC comprises a group of biologically similar cancers that start in the lip, oral cavity (mouth), nasal cavity (inside the nose), paranasal sinuses, pharynx, and larynx. Squamous cell carcinoma is the most common histological type of head and neck cancer, accounting for 90% of all head and neck malignancies. Gal-1 is highly overexpressed and secreted into the surrounding milieu by HNC [43,44]. Chawla et al. found that moderate to marked lymphocyte infiltrates were present in 58.8% of the HNC patient cohort, including T cells, B cells, and FoxP3-expressing T cells, while Gal-1 staining within lymphocyte areas of the tumor was significantly associated with poorer patient outcomes [45]. Nambiar et al. further found that patients with high tumoral or stromal Gal-1 expression had worse treatment responses and overall survival when treated with immune checkpoint inhibitors than those with low Gal-1 expression [46]. In addition, they showed that tumor-secreted Gal-1 inducted the transformation of tumor endothelium into an immune-suppressive barrier, preventing T cell migration into the tumor. Mechanistically, tumor-secreting Gal-1 reprograms the tumor endothelium to upregulate cell-surface programmed death-ligand 1 (PD-L1) and galectin-9, resulting in the inhibition of T cell infiltration [47]. Moreover, they showed that combining Gal-1 blockade with radiotherapy significantly improves the response to anti-PD1 immunotherapy [47].

### 4.3. Glioblastoma (GBM)

GBM is the most frequent and malignant human brain tumor, accounting for ∼50% of all primary brain tumor cases in adults [48]. Gal-1 is expressed in all types of human glioma [49,50,51]. Verschuere et al. showed that the silencing of glioma-derived Gal-1 significantly decreased the amount of brain-infiltrating macrophages and myeloid-derived suppressor cells (MDSC) in an orthotopic GL261 mouse glioma model [52]. In addition, they observed that the silencing of glioma-derived Gal-1 boosts IFN-ɣ production in the brain-infiltrating CD8+ T cells of tumor-bearing mice. Furthermore, they showed that the silencing of tumor-derived Gal-1 reduced vascular density and improved the outcomes of DC-vaccinated tumor-bearing mice. Chen et al. recently identified eight glioma microenvironmental genes from glioma databases (TCGA, CGGA, Rembrandt, GSE16011 and GSE43378) and discovered a key immunosuppressive gene, *LGALS1*, which obviously exhibited prognostic significance among glioma microenvironmental genes. In addition, they showed that the knockdown of *LGALS1* inhibits the GBM immunosuppressive microenvironment by down-regulating M2 macrophages and MDSC cells and by decreasing immunosuppressive cytokines such as CCL2, VEGFA, and TGF-β [53].

### 4.4. Pancreatic Ductal Adenocarcinoma (PDAC)

PDAC is an extremely aggressive malignancy and is resistant to currently available systemic therapies. Most PDAC is characterized by a prominent oncogenic tumor–stroma reaction around tumor tissue [54]. Pancreatic stellate cells (PSCs), which are stellate-shaped mesenchymal pancreatic cells, are one of the entities in the PDAC stroma. PSCs have been identified as important regulators of desmoplasia in PDAC [55]; Tang et al. showed that Gal-1 is expressed in abundance in activated PSCs. PSCs that overexpressed Gal-1 significantly induced the apoptosis of CD4^+^ T cells and CD8^+^ T cells and increased Th2 cytokine secretion (IL-4 and IL-5) from T cells [55]. Qian et al. demonstrated that Gal-1 induces the secretion of stromal cell-derived factor-1 (SDF-1) in PSCs, leading to increases in the migration and invasion of pancreatic cancer cells [56]. Martínez-Bosch et al. showed that the depletion of Gal-1 reduces the in vivo tumorigenicity, leading to significantly increased survival in the Ela-myc mouse pancreatic cancer model [21]. Mechanistically, Gal-1 activates the Hedgehog signaling pathway in PDAC epithelial and fibroblastic cells [21]. In a recent study by Orozco et al., the genetic deletion of Gal-1 decreased stroma activation, attenuated vascularization, and enhanced T cell infiltration in Kras-driven mouse pancreatic cancer models [57].

### 4.5. Lung Cancers

Galectin-1-expressing lung tumors have been connected to poor prognosis [58]. Chung et al. found that Gal-1 was overexpressed in non-small-cell lung cancer (NSCLC) cell lines [59]. Gal-1 could enhance the expression of COX-2, and its metabolite prostaglandin E2 (PGE2), to promote tumor progression in lung cancer [59]. The knockdown of Gal-1 in lung adenocarcinoma reduced tumor growth in vivo and inhibited cancer migration, invasion, and colony formation in vitro [59]. In research by Carlini et al., the immunohistochemical results showed that the expression of Gal-1 was detected in tumor cells, stroma, and blood vessels with positively stained endothelium in the tumor and surrounding normal tissue [22]. Kuo et al. showed that Gal-1 is highly expressed in the serum and surgical samples from lung cancer patients [60]. Functionally, Gal-1 was able to cause changes in the functions of monocyte-derived dendritic cells (MdDCs) by an IL-10 autocrine effect, which was regulated in an inhibitor of DNA binding 3(Id3)-dependent manner [60]. Hsu et al. showed that lung cancer-associated fibroblast (CAFs) are critical for the immunosuppression of TME by impairing the differentiation and function of dendritic cells (DCs) in lung cancer [61]. The immunosuppressive effect of CAF is mediated by elevated levels of the tryptophan 2,3-dioxygenase (TDO2)/kynurenine axis, which are triggered by lung cancer-derived Gal-1 [61].

### 4.6. Breast Cancer

Breast cancer develops in breast cells and most breast cancers form in the lobules or the ducts. Dalotto-Moreno et al. found that the expression of Gal-1 correlates with the aggressiveness of human breast tumors and is upregulated in the mouse metastatic 4T1 breast cancer model [62]. The inhibition of Gal-1 expression prevented tumor growth and suppressed the development of lung metastasis. In addition, they showed that tumor-derived Gal-1 promotes an immunosuppressive breast cancer microenvironment by increasing the frequency of CD4^+^CD25^+^ Foxp3^+^ Treg cells within the tumor, draining lymph nodes, spleen, and lung metastases [62]. Cheng et al. showed that tumor-derived Gal-1 could stimulate tolerogenic DCs differentiation after internalizing into CD14^+^ monocytes through the caveolae-dependent pathway and activating myosin IIa [63].

### 4.7. Melanomas

Melanomas have been proven to be resistant to apoptosis (type I programmed cell death) and, in effect, to chemotherapy and immunotherapy [64,65]. Cell death is most commonly associated with apoptosis, but it can also occur through other mechanisms, including autophagy. Rubinstein et al. identified Gal-1 as a major immunosuppressive factor secreted by human and murine melanoma cells. A blockade of the effects of Gal-1 within tumor tissue inhibited tumor growth and enhanced Th1-type antitumor response in syngeneic mice [66]. Yazawa confirmed this with results showing that melanoma cell adhesion molecule (MCAM) was one of the major melanoma cell Gal-1 ligands and was largely dependent on its N-glycans for Gal-1-binding [67]. Chemotherapy combinations were evaluated to improve clinical responses, but the overall survival (OS) rate did not show improvement. Mathieu et al. showed that decreasing Gal-1 expression in B16F10 mouse melanoma cells via siRNA sensitized cells to the anti-tumor effects of temozolomide in vivo but also induced heat shock protein 70-mediated lysosomal membrane permeabilization, a process associated with the release of cathepsin B into the cytosol, which in turn is believed to sensitize the cells to the pro-autophagic effects of temozolomide when grafted in vivo [68].

### 4.8. Neuroblastoma (NB)

Neuroblastoma (NB) is a very rare type of cancerous tumor that almost always affects children and develops from nerve cells in the fetus called neuroblasts. Sitek et al. showed that Gal-1 mRNA was upregulated in patients with aggressive and relapsing NB [69]. Consistent with their findings, Soldati et al. found that mouse and human NB cells expressed and secreted Gal-1, and that NB-derived soluble Gal-1 induced T cell apoptosis and inhibited DC maturation [70]. A NB murine model has been established by the targeted expression of the human MYCN oncogene in neuroectodermal cells under the control of rat *tyrosine hydroxylase* promoter (TH-MYCN) [71]. Büchel et al. further investigated the effect of Gal-1 on tumor formation, angiogenesis, and tumor–host interaction by cross-breeding Gal-1^−/−^ mice to TH-MYCN transgenic mice [72]. They found that TH-MYCN/Gal-1^−/−^ double transgenic mice displayed impaired tumor angiogenesis, splenomegaly, and impaired T cell tumor-infiltration, with no differences in T cell activation or apoptosis rate [72]. In addition, they observed that Gal-1^−/−^ CD4+ T cells had a lower migratory capacity toward tumor cells in vitro [72]. The transplantation of TH-MYCN-derived tumor cells into syngeneic mice resulted in significantly reduced tumor growth and elevated immune cell infiltration when Gal-1 was downregulated by shRNA [72]. These findings suggest the different effects of tumor- and immune cell-produced Gal-1: T cell-derived Gal-1 inhibits T cell tumor-infiltration, whereas NB-derived Gal-1 promotes tumor growth [72].

## 5. Therapeutic Agents against Gal-1 Signaling

**Figure 2:**
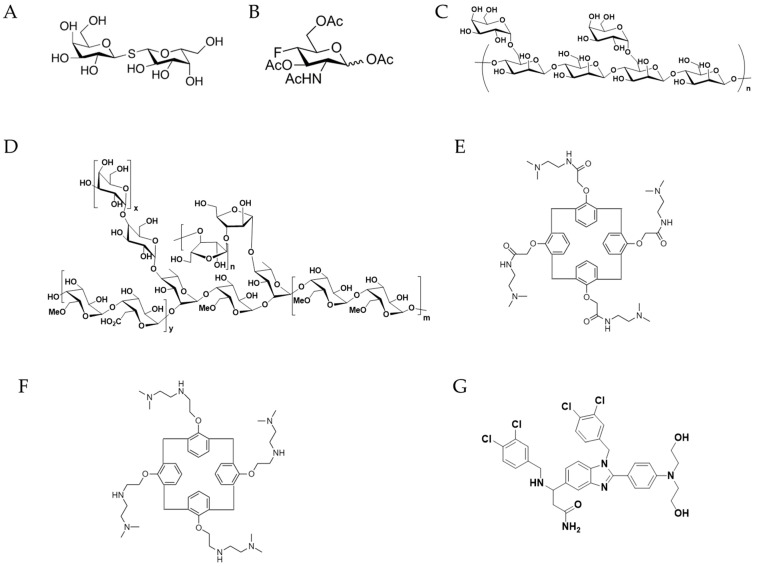
Chemical structures of Gal-1 inhibitors. (**A**) Thiodigalactoside. (**B**) 4-F-GlcNAc. (**C**) GM-CT-01. (**D**) GR-MD-02. (**E**) OTXOO8. (**F**) PTX013. (**G**) LLS30.

Given the important role of Gal-1 in tumor progression, targeting the Gal-1/ligand interaction represents a potential cancer therapeutic approach (Table 1). Stannard et al. showed that Gal-1 inhibition by thiodigalactoside (Figure 2A), a disaccharide, has proven effective in decreasing breast cancer progression when co-administered with vaccine immunotherapy [73,74,75,76]. Cedeno-Laurent et al. showed that limiting Gal-1-binding to LacNAc on T cell membrane proteins with peracetylated 4-fluoro-glucosamine (4-F-GlcNAc) (Figure 2B), a metabolic inhibitor of LAcNAc biosynthesis, decreased the growth of B16 melanomas and EL-4 lymphomas. 4-F-GlcNAc inhibitory efficacy on melanoma growth is driven by higher levels of anti-melanoma CTLs and lower levels of IL-10 [77]. Additionally, another strategy that has been developed is the administration of a DNA aptamer targeting Gal-1. Tsai et al. developed a Gal-1-targeting aptamer, AP-74 M-545, using the traditional SELEX (Systematic Evolution of Ligands by EXponential enrichment) method [78]. In addition, they showed that AP-74 M-545 binds to human and mouse Gal-1, leading to T cell apoptosis restoration and tumor growth inhibition [78].

Galectin Therapeutics Inc. has developed two such polysaccharides, GM-CT-01 (Davanat; Figure 2C) and GR-MD-02 (Belapectin; Figure 2D), which have been tested in clinical trials. In addition to binding to Gal-1, both GM-CT-01 and GR-MD-02 bind to Gal-3 [79]. GM-CT-01 has been tested alone and in combination with the chemotherapy drug 5-Fluorouracil (5-FU) in pre-clinical trials in Phase I and Phase II of clinical studies for metastatic colorectal cancer (ClinicalTrials.gov: NCT00110721 and NCT00054977) [80]. The clinical results show that: (a) GM-CT-01 was non-toxic, and a dose-limiting toxicity was not reached; (b) 70% of the patients were stabilized at the highest GM-CT-01 dose level (280 mg/m2/day) level; (c) a 46% increase in longevity of the patients (based on the Median Overall Survival) was achieved compared with the best standard of care; and (d) a 41% reduction in serious adverse effects was achieved compared to the best standard of care. In Phase II clinical studies, GR-MD-02 showed significant and clinically meaningful effects in nonalcoholic steatohepatitis (NASH) cirrhosis patients without esophageal varices (ClinicalTrials.gov: NCT02462967) [81]. There is an ongoing Phase I clinical study designed for a dose escalation of GR-MD-02, with the standard therapeutic dose of anti-PD1 (pembrolizumab) in patients with advanced melanoma, non-small-cell lung cancer, and head and neck squamous cell cancer (ClinicalTrials.gov: NCT02575404). OTX008 (Figure 2E), a small molecule Gal-1 inhibitor developed by Oncoethix, was tested in Phase I of the clinical studies for advanced solid tumors (ClinicalTrials.gov: NCT01724320). However, no reported outcomes of this clinical trial have been released. PTX013 (Figure 2F) modified from OTX008 is more potent at inhibiting the growth of several human cancer cell lines, as well as the growth of drug-resistant cancer cells [82].

**Table 1:** Applications of Gal-1 inhibitors in anticancer therapy.

Inhibitors	Materials	Disease	Effect	Study Trials	Ref
Thiodigalactoside	Disaccharides	Breast cancer	Inhibition of tumor growth. Synergistic effect with immunotherapy.	Rat	[73]
Peracetylated 4-fluoro-glucosamine (4-F-GlcNAc)	Glycan	B16 melanomas and EL-4 lymphomas	Inhibition of tumor growth. Elicit anti-melanoma CTLs and lower levels of IL-10.	Mouse	[77]
AP-74 M-545	Aptamer	Lewis lung carcinoma	T cell apoptosis restoration and tumor growth inhibition	Mouse	[78]
GM-CT-01 (Davanat) and/or 5-Fluorouracil	Polysaccharide and/or chemotherapeutic chemicals	Metastatic colorectal cancer	Enhancement in longevity of the patients and reduction in serious adverse effects.	Pre-clinical in Phase I and Phase II	[80] ClinicalTrials.gov: NCT00110721 and NCT00054977
GR-MD-02 (Belapectin)	Polysaccharide	NASH cirrhosis patients	Significant treatment effects in NASH cirrhosis patients without esophageal varices.	Phase II clinical trial	[81] ClinicalTrials.gov: NCT02462967
GR-MD-02 (Belapectin) with pembrolizumab	Polysaccharide and antibody respectively	Patients with advanced melanoma, non-small cell lung cancer, and head and neck squamous cell cancer	---	Phase I clinical trial	ClinicalTrials.gov: NCT02575404
OTX008	Small molecule	Advanced solid tumors	---	Phase I of the clinical studies	ClinicalTrials.gov: NCT01724320
PTX013	Small molecule	Human cancer cell lines and drug resistant cancer cells	Strong inhibitory effect on human cancer cell lines and drug-resistant cancer cells.	Cancer cells and drug- resistant cancer cells	[82]
LLS30	Small molecule	Human metastatic castration-resistant prostate cancer	Increasing the anti-tumor effect of docetaxel and inhibiting the invasion and metastasis of prostate cancer cells in vivo.	Castration-resistant prostate cancer xenograft	[83]

## 6. Conclusions

In this review, we have summarized the role of tumor-derived Gal-1 in tumor immune escape. In addition, a large number of studies have demonstrated the effect of Gal-1 on cancer progression and metastasis. Currently, no FDA-approved Gal-1 targeting agents are available in clinics, despite convincing experimental and pre-clinical data supporting the clear role of Gal-1 in cancer progression. Additional research on the development of Gal-1-targeting therapeutics is needed. We have developed a novel small-molecule Gal-1 inhibitor, LLS30 (Figure 2G), which is effective in treating prostate cancer in xenograft mouse models [83]. Additionally, LLS30 potentiates the antitumor effect of docetaxel and leads to a complete regression of PC3 castrate-resistant prostate cancer cells in vivo [83]. Further studies investigating the combined effects of LLS30 and anti-PD-1 therapy are currently ongoing (unpublished).

Cancer checkpoint blockade immunotherapy is a promising treatment for many forms of cancer. However, the response rates remain at only about 40% for melanoma [84,85], 25% for non-small-cell lung cancer [86], and <10% for most other cancer types [87]. Tumors with poor T cell trafficking are one of the challenges for checkpoint blockade cancer immunotherapy [88]. The mechanistic studies discussed in this review revealed that Gal-1 contributes to an immunosuppressive tumor microenvironment by inducing apoptosis in effector T cells. Thus, the manipulation of the Gal-1 signaling pathways provides a new avenue for improving checkpoint blockade immunotherapy outcomes. Indeed, in a recent study by Nambiar et al., Gal-1 blockade significantly increased intratumoral T cell infiltration, leading to a better response to anti-PD1 therapy in a HNC orthotopic tumor model [46]. Hence, the application of Gal-1 targeting deserves attention.
